# MACULAR NEURODEGENERATIVE AND VASCULAR CHANGES ON OPTICAL COHERENCE TOMOGRAPHIC ANGIOGRAPHY IN SICKLE CELL DISEASE ARE NOT RELATED TO ITS OCULAR AND SYSTEMIC COMPLICATIONS

**DOI:** 10.1097/IAE.0000000000004247

**Published:** 2024-11-12

**Authors:** Rajani P. Brandsen, Bart J. Biemond, Gulsum Z. Nasim, Erfan Nur, Reinier O. Schlingemann, Roselie M.H. Diederen

**Affiliations:** Departments of * Ophthalmology, and; † Hematology, Amsterdam UMC, Amsterdam, The Netherlands;; ‡ Sanquin Research and Landsteiner Laboratory, Department of Blood Cell Research, Amsterdam, The Netherlands; and; § Department of Ophthalmology, University of Lausanne, Jules-Gonin Eye Hospital, Fondation Asile des Aveugles, Lausanne, Switzerland.

**Keywords:** OCTA, macular abnormalities, sickle cell disease, sickle cell maculopathy, sickle cell retinopathy

## Abstract

**Purpose:**

To evaluate macular abnormalities in sickle cell disease with optical coherence tomographic angiography and to determine associations with sickle cell retinopathy (SCR) and clinical and laboratory characteristics.

**Methods:**

Complete ophthalmic examination was performed in consecutive patients with sickle cell disease (HbSS, HbSC, HbSβ^0^, or HbSβ^+^ genotype), including fundoscopy and macular spectral-domain optical coherence tomography/optical coherence tomographic angiography scans. Sickle cell retinopathy stage was based on fundoscopic examination (without fluorescein angiography) instead of the Goldberg classification because fluorescein angiography was only used in case of tentative diagnosis. Medical/ophthalmological history and hematologic characteristics were retrieved from medical records.

**Results:**

Two hundred and forty-nine eyes of 137 patients were analyzed. The mean age was 33.3 ± 12.4 years (range 15–70 years). Nonproliferative SCR was present in 57 eyes (22.9%) and proliferative SCR in 36 eyes (14.5%). Macular thinning was present in 100 eyes (40.2%) and was associated with lower foveal vessel density of the superficial capillary plexus and deep capillary plexus and with enlargement of the foveal avascular zone area, perimeter, and acircularity index. Age and female sex were associated with lower (para)foveal vessel density in the superficial capillary plexus and deep capillary plexus. No associations were found between SCR presence/severity and macular thinning or vessel density.

**Conclusion:**

Macular abnormalities were common but did not result in visual impairment. No relation with SCR presence/severity was found. Although optical coherence tomographic angiography imaging is suitable for detecting maculopathy, it appears to have no diagnostic value in identifying patients at risk for SCR.

## Abstract 2

Maculopathy on optical coherence tomographic angiography did not result in visual impairment, and no relation with sickle cell retinopathy presence or severity was found. Optical coherence tomographic angiography might be unsuitable for identifying patients at risk for peripheral retinopathy or other sickle cell disease–related organ damage, but it does point at different pathogenetic mechanisms at play in maculopathy and peripheral retinopathy.

Sickle cell disease (SCD) is a hereditary hemoglobinopathy, characterized by polymerization of abnormal hemoglobin upon deoxygenation. This polymerization results in chronic hemolytic anemia and recurrent microvascular occlusion, leading to ischemia and organ damage. Sickle cell retinopathy (SCR) is the most common ocular complication of SCD and occurs mainly in patients with the HbSC genotype (compound heterozygous SCD), which is associated with less severe systemic disease than the HbSS genotype (homozygous SCD).^1^ Advanced stages of SCR may cause vitreous hemorrhage (VH) or retinal detachment, which can drastically impair visual acuity. Although SCR is classically considered a peripheral retinopathy, several studies also reported macular changes since the early 1970s,^2^ but whether these are related to the peripheral changes has remained unclear.

Macular changes were reported using fluorescein angiography in approximately 32% of adult patients with homozygous SCD and in 36% of those with compound heterozygous SCD.^3^ More recently, imaging techniques such as spectral-domain optical coherence tomography (OCT) and OCT-angiography (OCTA) revealed subclinical macular changes in patients with asymptomatic SCD, which are not observed with traditional imaging techniques.^4,5^ Macular thinning was reported to be correlated with more advanced peripheral changes, suggesting a common pathophysiologic mechanism, and it was therefore proposed that macular thinning may be valuable as a surrogate marker in the clinical diagnosis of peripheral retinopathy.^6^

However, the functional consequences of the neurodegenerative changes leading to macular thinning are unclear. Scotomas on visual fields, reduced contrast sensitivity, and loss of color vision were reported in SCD patients with severely diminished vessel density (VD) on OCTA, even when the visual acuity was unaffected.^7^ Irreversible visual function loss might therefore occur secondary to both sickle cell maculopathy and proliferative sickle cell retinopathy (PSCR). Furthermore, macular abnormalities may be of potential interest as a proxy of vascular abnormalities in other organs as a clinical biomarker or to help understand the pathogenesis of vascular changes in SCD.

This study aims to analyze the frequency and severity of macular thinning and macular perfusion abnormalities detected by OCTA in a large cohort of adult patients with SCD and to assess their possible associations with SCR presence or severity and other clinical characteristics.

## Methods

This single-institution cross-sectional study was conducted by a collaboration between the Departments of Ophthalmology and Hematology of the Amsterdam University Medical Centers, Amsterdam, The Netherlands. Consecutive patients with SCD (HbSS, HbSβ^0^, HbSβ^+^, and HbSC) visiting the adult outpatient SCD clinic were referred to the Department of Ophthalmology if there was no prior ophthalmic examination at our center or if their last examination was more than three years ago. Patients with hereditary or acquired retinopathy (e.g., diabetic retinopathy [DR], retinal vascular occlusions), any ocular media opacities preventing detailed imaging, high myopia (>6 diopters), or vitreomacular interface abnormalities were excluded. The study was approved by the Institutional Review Board of the Amsterdam University Medical Centers and carried out in accordance with the principles of the Declaration of Helsinki (seventh revision, 2013). Verbal consent from participants was obtained.

Complete ophthalmic examination was performed, including assessment of Snellen distance best-corrected visual acuity, slit-lamp examination of the anterior segment, and dilated fundus biomicroscopy. Visual impairment was defined as a visual acuity below 20/25. Fluorescein angiography, which is an essential element of the commonly used Goldberg classification,^8^ was not systematically performed but was only used in case of tentative diagnosis of PSCR or for patients with Stage III disease or worse. Therefore, SCR stage was determined for each eye in this study based on fundoscopic examination without fluorescein angiography (instead of the Goldberg classification) as follows: 1) no signs of SCR on fundoscopic examination; 2) signs of nonproliferative SCR (NPSCR) on fundoscopic examination (sunburst lesions, salmon patches, arteriolar occlusions, peripheral anastomose without neovascularization); or 3) proliferative SCR on fundoscopic examination (with neovascularization). Eyes with previously resolved VH and treated retinal detachment were also classified as PSCR, respectively.

**Fig. 1.**
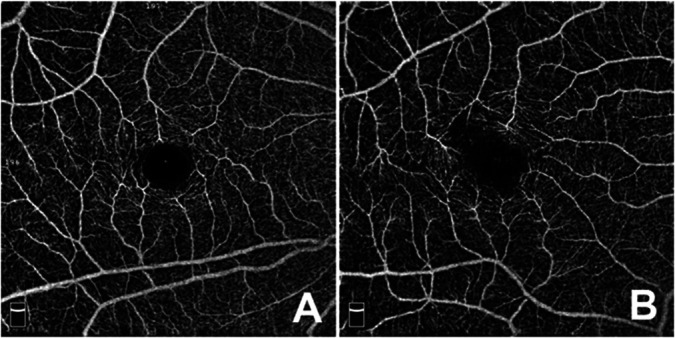
Normal and abnormal OCTA scan. **A.** Normal OCTA scan. **B.** Abnormal OCTA scan with asymmetrical enlargement of the FAZ.

**Fig. 2.**
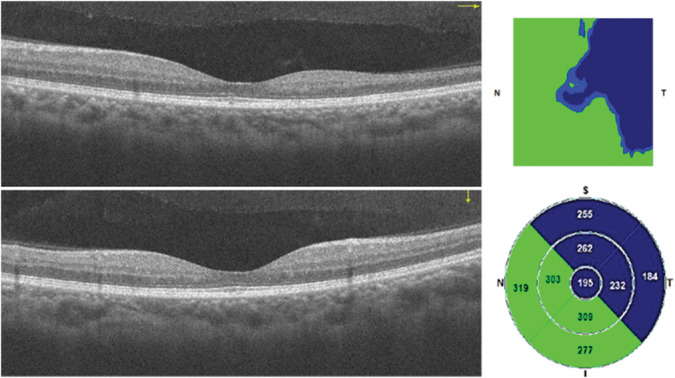
Abnormal B scan. Pathological macular thinning is present in the superior and temporal subfields.

Macular spectral-domain optical coherence tomography and OCTA scans were obtained with the Optovue RTVue XR Avanti instrument (Optovue Inc, Fremont, CA). For each eye, a 3 mm × 3 mm and a 6 mm × 6 mm scan centered on the fovea were acquired. AngioAnalytics software (beta version 2016.200.037) for projection artifact removal and automated segmentation of the superficial capillary plexus (SCP) and deep capillary plexus (DCP) was used. In addition, the VD in the SCP and DCP as well as foveal avascular zone (FAZ) measurements (area, perimeter and acircularity index [AI]) were automatically calculated (see Figure 1 for examples of a normal [1A] and abnormal [1B] OCTA image). The retinal thickness color-coded map was qualitatively evaluated for patchy blue areas of retinal thinning to assess the presence of macular thinning. Macular thinning was defined as the presence of blue areas in at least one of the standard Early Treatment Diabetic Retinopathy Study macular subfields (Figure 2). The blue color signifies a probability value of less than 1%, indicating that less than 1% of normal eyes exhibit values as low as those detected. Images were excluded if the quality index was four or lower and if motion artifacts limited adequate grading. The automated segmentation lines and correct centration of the Early Treatment Diabetic Retinopathy Study circle were manually checked to ensure accuracy.

Available ophthalmological data and hematologic characteristics were retrieved from the electronic patient records, including Hb genotype, use of hydroxyurea, presence of microalbuminuria, avascular necrosis, history of acute chest syndrome, stroke, chronic ulcers, cholelithiasis, and laboratory characteristics (Hb, creatinine, LDH, bilirubin, reticulocytes). Microalbuminuria was defined as a urinary microalbumin (mg/L) to creatinine (mmol/L) ratio of more than 3.5. Avascular necrosis had to be confirmed by x-ray or MRI. Acute chest syndrome was defined as the finding of a new pulmonary infiltrate on chest radiography in combination with fever, respiratory symptoms, or chest pain for which medical treatment was necessary. Stroke was defined as a diagnosis of clinical overt stroke confirmed by MRI or CT. Chronic ulcers are defined as chronic ulceration of the lower leg, without another explanation, that did not heal within 14 days. Cholelithiasis was defined as the presence of gallstones (demonstrated by ultrasound) or a previous cholecystectomy because of cholecystolithiasis.

Statistical analysis was performed using IBM SPSS Statistics (Version 28.0; IBM Corp, Armonk, NY) and the R software environment (version 4.2.0; R Foundation for Statistical Computing, Vienna, Austria). Completeness of data was checked before performing the analysis. Cases with missing data on used OCTA parameters were excluded. A small amount of missing data in laboratory parameters was imputed using single imputation. Binary and categorical variables are displayed as frequencies and percentages. Continuous variables are displayed as means with SDs. Logistic and linear mixed models with random intercept for cases were used to test differences between the groups and calculate odds ratios (ORs), while considering interocular correlation. For the calculation of ORs and estimates, values of HbF, LDH, and bilirubin were converted to the natural log scale. Measures of SVD and DVD were scaled by a 1/10 factor. Foveal avascular zone, perimeter, and AI were scaled to SD = 1 with scales 0.129, 479.114, and 0.051, respectively. *P* < 0.05 were considered significant. Given *P* values are uncorrected due to the exploratory character of this study.

## Results

A total of 278 eyes of 144 consecutive patients with SCD were enrolled between November 2017 and April 2022. OCTA scans with a quality index of four or lower (n = 21 eyes) and scans with a nonmeasurable FAZ area due to artifacts (n = 8 eyes) were excluded. This resulted in 249 eyes (125 right eyes; 124 left eyes) of 137 included patients. Baseline characteristics are outlined in Table 1. The mean age was 33.3 years (SD ± 12.4, range 15–70 years), and the numbers of male and female patients were similar (49.6% vs. 50.4%). Most patients had the HbSS genotype (48.9%), followed by HbSC genotype (35.8%), HbSβ^+^ (10.2%), and HbSβ^0^ (5.1%). Nonproliferative SCR was present in 57 eyes (22.9%) and PSCR in 36 eyes (14.5%). Hydroxyurea was used by 45 patients (32.8%).

**Table 1.** Baseline Characteristics and Stratification by Macular Thinning

	Total	No Macular Thinning	Macular Thinning	*P* *
Patients, n (%)	137 (100)	81 (59.1)	56 (40.9)	—
Eyes, n (%)	249 (100)	149 (59.8)	100 (40.2)	—
OD, n eyes (%)	125 (50.2)	76 (51)	49 (49)	
OS, n eyes (%)	124 (49.8)	73 (49)	51 (51)	
Gender				
Male, n (%)	68 (49.6)	44 (54.3)	24 (42.9)	**0.044**
Female, n (%)	69 (50.4)	37 (45.7)	32 (57.1)	
Age (years)	33.3 ± 12.4	32.2 ± 12.1	34.8 ± 12.7	0.065
Hb genotype				
HbSS, n (%)	67 (48.9)	36 (44.4)	31 (55.4)	0.206
HbSC, n (%)	49 (35.8)	35 (43.2)	14 (25)	
HbSβ^0^, n (%)	7 (5.1)	4 (4.9)	3 (5.4)	
HbSβ^+^, n (%)	14 (10.2)	6 (7.4)	8 (14.3)	
Retinopathy				
None, n eyes (%)	156 (62.7)	98 (65.8)	58 (58)	0.090
NPSCR, n eyes (%)	57 (22.9)	27 (18.1)	30 (30)	
PSCR, n eyes (%)	36 (14.5)	24 (16.1)	12 (12)	
VA < 20/25, n eyes (%)	6 (2.4)	5 (3.4)	1 (1)	0.951
Ocular complications				
VH, n eyes (%)	11 (4.4)	9 (6)	2 (2)	**0.035**
RD, n eyes (%)	4 (1.6)	1 (0.7)	3 (3)	0.105
Laser treatment, n eyes (%)	21 (8.4)	11 (7.4)	10 (10)	0.292
Hydroxyurea use, n (%)	45 (32.8)	28 (34.6)	17 (30.4)	0.186
Voxelotor use, n (%)	12 (8.8)	8 (9.9)	4 (7.1)	0.160
Crizanlizumab use, n (%)	1 (0.7)	0 (0)	1 (1.8)	^†^
Chronic transfusion use, n (%)	7 (5.1)	4 (4.9)	3 (5.4)	0.450
Laboratory characteristics				
Hb (g/dL)	10.2 ± 2.1	10.7 ± 2	9.6 ± 2	**0.003**
HbF (%)	6.1 ± 6.2	6.0 ± 6.4	6.3 ± 5.9	0.650
Creatinine (*µ*mol/L)	69.5 ± 19.7	68.2 ± 17.3	71.4 ± 22.9	0.579
LDH (U/L)	366.8 ± 190.9	337.1 ± 180.1	409.8 ± 199.4	0.194
Bilirubin (*µ*mol/L)	43.5 ± 56.8	40.2 ± 34.8	48.3 ± 78.5	0.818
Reticulocytes (%)	6.6 ± 4.7	6.1 ± 4.1	7.4 ± 5.3	0.092
Other organ damage				
Microalbuminuria, n (%)	35 (25.5)	21 (25.9)	14 (25)	0.989
AVN, n (%)	21 (15.3)	13 (16)	8 (14.3)	0.645
ACS, n (%)	43 (31.4)	26 (32.1)	17 (30.4)	0.801
Stroke, n (%)	7 (5.1)	5 (6.2)	2 (3.6)	0.610
Chronic ulcer, n (%)	6 (4.4)	3 (3.7)	3 (5.4)	0.893
Cholelithiasis, n (%)	52 (38)	31 (38.3)	21 (37.5)	0.989
OCTA parameters (mean ± SD)				
Foveal VD SCP (%)	18.66 ± 7.90	20.14 ± 7.29	16.45 ± 8.29	**0.020**
Parafoveal VD SCP (%)	53.36 ± 4.97	54.30 ± 3.61	51.97 ± 6.24	**0.010**
Perifoveal VD SCP (%)	52.21 ± 3.80	52.98 ± 3.34	51.06 ± 4.14	**0.001**
Foveal VD DCP (%)	33.85 ± 8.81	35.82 ± 7.42	30.92 ± 9.89	**0.001**
Parafoveal VD DCP (%)	55.70 ± 4.30	55.48 ± 4.14	56.02 ± 4.53	0.920
Perifoveal VD DCP (%)	54.32 ± 5.88	54.39 ± 6.01	54.20 ± 5.72	0.470
FAZ area (mm^2^)	0.353 ± 0.129	0.326 ± 0.106	0.392 ± 0.149	**0.010**
FAZ perimeter (mm)	2.29 ± 0.48	2.19 ± 0.41	2.44 ± 0.53	**0.001**
FAZ AI	1.11 ± 0.05	1.10 ± 0.04	1.13 ± 0.06	**0.001**

Significance for bold entries was *P* < 0.05.

* Logistic mixed model with random intercept for cases.

† An estimate for crizanlizumab could not be obtained as only one case received this (perfect separation).

ACS, acute chest syndrome; AVN, avascular necrosis; RD, retinal detachment.

### Macular Thinning on Optical Coherence Tomography

The baseline characteristics stratified by the presence of macular thinning are shown in Table 1. Macular thinning was present in 100 eyes (40.2%). The temporal macular region was predominantly affected with thinning (76% of the affected eyes). Macular thinning was present in the superior macula in 48% of the affected eyes, in the inferior macula in 46%, and in the nasal macula in 44%. Thinning in the central subfield was present in 39% of the affected eyes.

Macular thinning was more prevalent in female patients than in male patients (*P* = 0.044). A clear association was found between macular thinning and the absence of VH (*P* = 0.035) and a lower hemoglobin level (*P* = 0.003). No significant association between macular thinning and the presence/severity of SCR was demonstrated (OR 0.93, 95% confidence interval [CI] 0.13–6.78, *P* = 0.09). Although there was a trend for more HbSS patients than HbSC patients in the macular thinning group (*P* = 0.206), stratification by genotype (HbSC vs. HbSS/HbSβ-thalassemia) did not reveal an association between macular thinning and SCR (HbSC: OR −2.41, 95% CI: −2.03 to 4.15, *P* = 0.72, other genotypes: OR −0.79, 95% CI: −2.07 to 7.20, *P* = 0.35). On OCTA, in patients with macular thinning, the VD of the SCP was lower in the foveal (16.45 ± 8.29 vs. 20.14 ± 7.29, *P* = 0.02), parafoveal (51.97 ± 6.24 vs. 54.30 ± 3.61, *P* = 0.01), and perifoveal (51.06 ± 4.14 vs. 52.98 ± 3.34, *P* = 0.001) subfields compared with patients without macular thinning. Similarly, the foveal VD of the DCP was lower in patients with macular thinning (30.92 ± 9.89 vs. 35.82 ± 7.42, *P* = 0.001). Moreover, this group had a larger FAZ area (0.392 ± 0.149 vs. 0.326 ± 0.106, *P* = 0.01), FAZ perimeter (2.44 ± 0.53 vs. 2.19 ± 0.41, *P* = 0.001), and AI (1.13 ± 0.06 vs. 1.10 ± 0.04, *P* = 0.001). Other baseline characteristics did not differ between the groups with and without macular thinning.

### Other Optical Coherence Tomographic Angiography Measurements

Mean values for VD and FAZ parameters stratified by genotype groups (HbSS/HbSβ^+^/HbSβ^0^ vs. HbSC) are shown in Table 2. No differences between these groups were observed. Multivariate analysis of factors associated with VD parameters is shown in Table 3. Female patients had a lower foveal VD in the SCP and DCP compared with male patients (*P* = 0.008 and *P* = 0.025, respectively). Furthermore, older age was associated with a lower parafoveal VD in the SCP and DCP (*P* = 0.013 and *P* = 0.031, respectively). A borderline association was found between reticulocyte levels and parafoveal VD in the DCP (*P* = 0.05). Hb genotype and SCR presence/severity did not correlate with VD parameters.

**Table 2.** Optical Coherence Tomographic Angiography Measurements Stratified by Genotype

	HbSS/HbS-Thalassemia (n = 164 Eyes)	HbSC (n = 85 Eyes)	*P* *
Vessel density SCP (%, mean ± SD)			
Foveal	18.66 ± 8.23	18.66 ± 7.28	0.961
Parafoveal	53.46 ± 4.83	53.17 ± 5.23	0.918
Perifoveal	52.36 ± 3.65	51.92 ± 4.07	0.790
Vessel density DCP (%, mean ± SD)			
Foveal	33.99 ± 9.14	33.58 ± 8.17	0.433
Parafoveal	56.37 ± 4.14	54.40 ± 4.32	0.098
Perifoveal	54.73 ± 5.95	53.52 ± 5.70	0.199
FAZ (mean ± SD)			
Area (mm^2^)	0.352 ± 0.14	0.354 ± 0.12	0.953
Perimeter (mm)	2.29 ± 0.48	2.30 ± 0.49	0.888
AI	1.11 ± 0.05	1.11 ± 0.05	0.885

* Logistic mixed model with random intercept for cases.

**Table 3.** Multivariate Analyses VD

	Foveal Vessel Density SCP (Estimator in %, 95% CI)†	*P*	Parafoveal Vessel Density SCP (Estimator in %, 95% CI)†	*P*	Foveal Vessel Density DCP (Estimator in %, 95% CI)†	*P*	Parafoveal Vessel Density DCP (Estimator in %, 95% CI)†	*P*
Sex								
Male	Ref		Ref		Ref		Ref	
Female	**−0.391 (−0.656 to −0.124)**	**0.008**	0.061 (−0.097 to 0.218)	0.477	**−0.358 (−0.674 to −0.041)**	**0.039**	0.121 (−0.016 to 0.259)	0.110
Hb genotype								
HbSS/S-thal	Ref		Ref		Ref		Ref	
HbSC	−0.008 (−0.448 to 0.435)	0.973	0.093 (−0.171 to 0.357)	0.521	−0.073 (−0.595 to 0.451)	0.797	−0.097 (−0.327 to 0.133)	0.439
Age	−0.007 (−0.018 to 0.004)	0.246	**−0.008 (**−**0.014 to** −**0.001)**	**0.036**	−0.006 (−0.019 to 0.007)	0.396	**−0.006 (−0.012 to** −**0.001)**	**0.042**
SCR stage								
No SCR	Ref		Ref		Ref		Ref	
NPSCR	−0.171 (−0.388 to 0.069)	0.155	0.002 (−0.153 to 0.163)	0.977	−0.202 (−0.430 to 0.055)	0.107	0.004 (−0.135 to 0.132)	0.960
PSCR	−0.023 (−0.337 to 0.301)	0.890	−0.056 (−0.277 to 0.165)	0.638	−0.085 (−0.422 to 0.255)	0.633	−0.129 (−0.313 to 0.058)	0.192
Hb	−0.0004 (−0.159 to 0.158)	0.996	0.0007 (−0.093 to 0.095)	0.990	0.068 (−0.121 to 0.256)	0.506	0.011 (−0.071 to 0.093)	0.812
HbF*	0.068 (−0.087 to 0.223)	0.422	0.020 (−0.073 to 0.112)	0.693	0.044 (−0.140 to 0.229)	0.658	0.016 (−0.065 to 0.097)	0.710
LDH*	−0.071 (−0.362 to 0.220)	0.652	0.072 (−0.101 to 0.245)	0.444	−0.060 (−0.405 to 0.286)	0.748	0.063 (−0.087 to 0.215)	0.444
Bilirubin*	0.058 (−0.253 to 0.135)	0.581	−0.030 (−0.144 to 0.086)	0.633	0.053 (−0.178 to 0.282)	0.673	−0.073 (−0.173 to 0.028)	0.183
Reticulocytes	0.009 (−0.024 to 0.043)	0.604	0.017 (−0.003 to 0.037)	0.128	0.005 (−0.035 to 0.044)	0.834	0.019 (0.001 to 0.036)	0.050
Microalbuminuria	0.023 (−0.286 to 0.330)	0.893	−0.077 (−0.257 to 0.108)	0.440	−0.026 (−0.393 to 0.339)	0.895	0.004 (−0.156 to 0.164)	0.965
AVN	0.124 (−0.210 to 0.461)	0.496	0.025 (−0.175 to 0.224)	0.820	0.153 (−0.245 to 0.553)	0.479	−0.027 (−0.202 to 0.147)	0.778
ACS	0.081 (−0.181 to 0.342)	0.567	0.070 (−0.086 to 0.223)	0.409	0.043 (−0.268 to 0.353)	0.797	0.017 (−0.118 to 0.153)	0.815
Stroke	−0.174 (−0.747 to 0.400)	0.577	−0.149 (−0.489 to 0.192)	0.423	−0.101 (−0.782 to 0.579)	0.784	−0.144 (−0.443 to 0.152)	0.375
Chronic ulcers	0.573 (−0.049 to 1.199)	0.092	0.030 (−0.343 to 0.401)	0.882	0.582 (−0.156 to 1.323)	0.148	−0.027 (−0.353 to 0.296)	0.879
Cholelithiasis	−0.057 (−0.330 to 0.215)	0.699	−0.073 (−0.235 to 0.089)	0.412	0.041 (−0.281 to 0.365)	0.813	−0.049 (−0.191 to 0.092)	0.525

Significance for bold entries was *P* < 0.05.

* Units on Ln scale.

† Per 10 units.

ACS, acute chest syndrome; AVN, avascular necrosis.

The multivariate analysis of factors associated with FAZ area, FAZ perimeter, and FAZ AI is shown in Table 4. Female patients had a larger FAZ area than male patients (*P* = 0.033). The FAZ perimeter seemed also larger in female patients, but this finding was borderline nonsignificant (*P* = 0.058). There was no difference between female and male patients in AI. However, the AI was larger in NPSCR patients compared with patients without retinopathy (*P* = 0.026). This same trend was seen for PSCR but did not reach statistical significance. Regarding sickle cell–related organ damage, the AI was larger in patients with avascular necrosis (*P* = 0.037). Hb genotype, laboratory characteristics, and other forms of organ damage had no demonstrable correlations with the FAZ parameters.

**Table 4.** Multivariate Analyses FAZ Parameters

	FAZ Area (Estimator in mm^2^, 95% CI)†	*P*	FAZ Perimeter (Estimator in mm, 95% CI)‡	*P*	FAZ Acircularity Index (Estimator, 95% CI)§	*P*
Sex						
Male	**Ref**		Ref		Ref	
Female	**0.394 (0.042–0.745)**	**0.040**	0.335 (−0.002 to 0.669)	0.068	−0.025 (−0.322 to 0.265)	0.876
Hb genotype						
HbSS/S-thal	Ref		Ref		Ref	
HbSC	0.032 (−0.550 to 0.614)	0.919	0.167 (−0.391 to 0.722)	0.580	0.401 (−0.112 to 0.883)	0.145
Age	−0.0006 (−0.015 to 0.014)	0.935	0.002 (−0.012 to 0.016)	0.761	0.011 (−0.001 to 0.023)	0.110
SCR stage						
No SCR	Ref		Ref		Ref	
NPSCR	0.218 (−0.067 to 0.476)	0.124	0.245 (−0.051 to 0.518)	0.102	**0.372 (0.065 to 0.680)**	**0.026**
PSCR	0.118 (−0.274 to 0.489)	0.557	0.135 (−0.270 to 0.527)	0.521	0.322 (−0.114 to 0.750)	0.169
Hb	−0.080 (−0.288 to 0.130)	0.483	−0.077 (−0.276 to 0.124)	0.477	−0.142 (−0.314 to 0.038)	0.147
HbF*	−0.099 (−0.303 to 0.106)	0.375	−0.055 (−0.251 to 0.141)	0.608	0.077 (−0.101 to 0.247)	0.422
LDH*	0.092 (−0.293 to 0.475)	0.659	0.119 (−0.249 to 0.485)	0.551	0.352 (0.030–0.675)	0.051
Bilirubin*	−0.015 (−0.271–0.241)	0.911	−0.007 (−0.251–0.238)	0.960	−0.036 (−0.253–0.177)	0.762
Reticulocytes	0.001 (−0.043 to 0.045)	0.958	0.001 (−0.041 to 0.043)	0.969	0.014 (−0.023 to 0.051)	0.508
Microalbuminuria	−0.030 (−0.436 to 0.378)	0.892	−0.038 (−0.425 to 0.352)	0.856	−0.207 (−0.549 to 0.134)	0.275
AVN	−0.231 (−0.675 to 0.211)	0.338	−0.103 (−0.529 to 0.318)	0.653	**0.435 (0.050–0.799)**	**0.037**
ACS	−0.047 (−0.391 to 0.299)	0.801	−0.009 (−0.338 to 0.321)	0.960	−0.044 (−0.330 to 0.248)	0.785
Stroke	0.112 (−0.645 to 0.869)	0.785	0.070 (−0.652 to 0.793)	0.858	−0.247 (−0.879 to 0.395)	0.483
Chronic ulcers	−0.661 (−1.483 to 0.161)	0.140	−0.551 (−1.335 to 0.237)	0.198	0.592 (−0.094 to 1.304)	0.127
Cholelithiasis	−0.144 (−0.503 to 0.216)	0.461	−0.131 (−0.474 to 0.213)	0.484	−0.105 (−0.403 to 0.204)	0.532

Significance for bold entries was *P* < 0.05.

* Units on Ln scale.

† On scale 0.129.

‡ On scale 479.114.

§ On scale 0.051.

ACS, acute chest syndrome; AVN, avascular necrosis.

## Discussion

This study evaluated macular abnormalities in a consecutive cohort of adult SCD patients using OCT and OCTA parameters and analyzed their associations with clinical and laboratory characteristics. To our knowledge, this is the largest study with OCTA in SCD patients to date. We demonstrated that macular thinning and OCTA abnormalities are common in SCD. No association could be demonstrated with the presence/severity of SCR or visual impairment.

Macular thinning in retinal and systemic neurodegenerative diseases is a widely reported phenomenon since the introduction of the OCT device and can be caused by various factors. In healthy eyes, macular thinning can be observed with increasing age due to age-related loss of retinal nerve fibers and ganglion cells.^9^ In patients with diseases such as diabetes mellitus and Alzheimer disease, significantly more pronounced macular thinning than in healthy populations has been described.^10,11^

Many OCTA studies have been conducted to study microvascular parameters in diabetic patients, demonstrating FAZ enlargement in Type 1 and Type 2 diabetes mellitus, which correlated with peripheral areas of ischemia and even progression of DR.^12,13^ A recent meta-analysis demonstrated acceptable specificity and sensitivity of the macular OCTA for diagnosing DR, including grading of severity.^14^ These findings suggest a common underlying pathogenesis leading to capillary nonperfusion in macular and peripheral regions in DR, although the results of our study did not demonstrate a relation between macular thinning, OCTA abnormalities, and SCR in SCD. In diabetic patients without any sign of retinopathy, structural changes in the FAZ area and reduction of VD were also present; but the changes were milder in diabetic patients than in patients with retinopathy.^15^ These findings emphasize the essential differences between the pathogenesis and clinical manifestations of DR and SCR.

Macular thinning was predominantly present in the temporal macular region, which aligns with previous studies.^16,17^ The retinal arterioles consist of fairly long sections with equal diameters throughout their course, which may result in relatively high vascular resistance. Because the terminal arterioles in the temporal macula are longer and have a smaller diameter than the other macular regions, this area might be more susceptible to microvascular obstruction and ischemia due to SCD or other causes.^2,18^ Furthermore, macular thinning was more prevalent among our female patients, which has not been described in previous studies. This finding contrasts with the prevalence of peripheral SCR in SCD, which is reportedly associated with male sex.^1,19^ Higher hemoglobin levels in male patients and a protective effect of estrogen on endothelial function in female patients are hypothesized to explain the lower prevalence of retinopathy in female patients.^19,20^ Although our female patients did not differ from male patients in distribution of Hb genotype or SCR severity, they did differ in age. The mean age in female patients was 36.8 ± 13.6 years versus 29.7 ± 9.9 years in male patients. This might explain our finding, as the prevalence of sickle cell maculopathy increases with age.^21^ Moreover, the overall macular thickness is generally lower in female patients than in male patients.^22^ Macular thinning was associated with the absence of VH and lower hemoglobin levels. This might be explained by the proportionally higher prevalence of the HbSS genotype in the group with macular thinning, as these patients have lower hemoglobin levels than HbSC patients, and PSCR (including VH) is less common among HbSS patients.^23^ The trend for higher prevalence of macular thinning in HbSS seems to emphasize its independence of the peripheral vascular abnormalities in SCR, as this disease has a higher prevalence in HbSC patients.

Interestingly, our study did not find a correlation between hydroxyurea use and macular thinning. This is in contrast with the study of Lim et al,^24^ which showed lower rates of macular thinning over time in patients using hydroxyurea. This discrepancy might be explained by the difference in the definition of macular thinning because our study measured this as “present” or “absent,” and the study from Lim et al measured this as a change in the macular thickness over time, whereas our analysis is cross sectional, lacking information on the effect of hydroxyurea over time. Furthermore, their population had a higher proportion of hydroxyurea users (51.4%) compared with ours (32.8%), which increases the power to detect an association with macular thinning. The difference in hydroxyurea use is likely related to the HbSS genotype prevalence (63.4% in their study vs. 48.9% in ours) and the fact that hydroxyurea is more frequently prescribed to patients with the HbSS genotype.

Our female patients had a significantly lower foveal VD in both the SCP and DCP and a larger FAZ area compared with male patients by multivariate analysis. Although previous studies on SCD did not demonstrate smaller FAZ areas in male patients, this finding has been reported in one study in the general population.^16^ Studies on OCTA parameters in healthy populations also showed lower vessel densities and larger FAZ areas in female patients, as well as in older patients.^25,26^ Therefore, our findings on lower vessel densities in female patients and older age might not be specific for SCD but are in line with the situation in the general population.

Sickle cell maculopathy and more profound loss of flow and VD correlated with PSCR in previous studies.^16,17,27^ By contrast, our study (with a large cohort of SCD patients studied) did not reveal any association between macular VD parameters and SCR presence or severity or Hb genotype. A notable difference between the previous studies and our present study is the prevalence of PSCR. However, given the high absolute number of patients with PSCR in our cohort, this seems not to be a likely explanation for this difference. Regarding Hb genotype, a higher proportion of HbSS patients did have macular thinning, but this did not reach statistical significance and analysis stratified by genotype did not reveal an association between macular thinning and SCR. Previous studies in pediatric SCD cohorts reported more extensive loss of flow in HbSS patients compared with compound heterozygous sickle cell patients.^28,29^ However, other studies in adult SCD cohorts did not report this association.^27,30^ Possibly, macular thinning has an earlier onset in patients with HbSS, which could explain these differences between pediatric and adult populations.

The findings of our study did not reveal a relation between sickle cell maculopathy and peripheral retinopathy. Patients with maculopathy were overall characterized by features more related to HbSS genotype (e.g., lower Hb), whereas patients with PSCR are generally characterized by features of the HbSC genotype. Therefore, we speculate that the underlying pathophysiology of sickle cell maculopathy might differ from that of peripheral retinopathy or that it might be different in HbSS and HbSC patients. The macula may be more susceptible to diffuse microvascular loss than the peripheral retina, where ongoing ischemia resulting in neovascularization is presumed to be the major problem.^31^ We further hypothesize that accelerated aging might play a role in sickle cell maculopathy because macular thinning, lower vessel densities, and larger FAZ areas are also associated with age in healthy populations. This phenomenon, where abnormalities that usually occur in the elderly are present in SCD patients at a significantly younger age, has previously been described for other SCD-related organ complications as “accelerated aging syndrome.”^32^ Furthermore, SCD related maculopathy seems to be present without impairing the visual acuity. However, its behavior over time (including development of visual symptoms) is unknown because longitudinal studies are lacking.

Our study had several limitations. Although the total patient cohort was large, PSCR was found in only a relatively small portion of the patients. In addition, the prevalence of Goldberg Stage I and II retinopathy might be underestimated as we did not routinely perform fluorescein angiography.^33^ When interpreting the results of our study, it should be taken into account that peripheral ischemia, which was associated with macular OCTA abnormalities in another study, was also not evaluated due to the lack of fluorescein angiography, which is not routinely performed in our clinic.^34^ Furthermore, we chose to keep *P* values uncorrected due to the exploratory character of this study. This might increase the error rate with multiple analyses.

In conclusion, we demonstrate that macular thinning and associated OCTA abnormalities are common in adult SCD patients. However, no relation between OCTA abnormalities and the presence or severity of SCR on fundoscopic examination could be demonstrated. Therefore, OCTA analysis seems not useful in identifying SCD patients at risk for peripheral SCR but does point at differential microvascular pathogenetic mechanisms at play in SCD maculopathy and SCR.
